# *GmPRP2* promoter drives root-preferential expression in transgenic *Arabidopsis* and soybean hairy roots

**DOI:** 10.1186/s12870-014-0245-z

**Published:** 2014-09-16

**Authors:** Li Chen, Bingjun Jiang, Cunxiang Wu, Shi Sun, Wensheng Hou, Tianfu Han

**Affiliations:** MOA Key Laboratory of Soybean Biology (Beijing), Institute of Crop Sciences, The Chinese Academy of Agricultural Sciences, Beijing, China

**Keywords:** Soybean, PRP2, Promoter, Root-preferential, *Arabidopsis thaliana*, Deletion, GUS, Hairy root

## Abstract

**Background:**

Promoters play important roles in gene expression and function. There are three basic types of promoters: constitutive, specific, and inducible. Constitutive promoters are widely used in genetic engineering, but these promoters have limitations. Inducible promoters are activated by specific inducers. Tissue-specific promoters are a type of specific promoters that drive gene expression in specific tissues or organs. Here, we cloned and characterized the *GmPRP2* promoter from soybean. The expression pattern indicated that this promoter is root-preferential in transgenic *Arabidopsis* and the hairy roots of soybean. It can be used to improve the root resistance or tolerance to pathogens, pests, malnutrition and other abiotic stresses which cause extensive annual losses in soybean production.

**Results:**

The *GmPRP2* promoter (GmPRP2p-1062) was isolated from soybean cv. Williams 82. Sequence analysis revealed that this promoter contains many *cis*-acting elements, including root-specific motifs. The GmPRP2p-1062 and its 5’-deletion fragments were fused with the *GUS* reporter gene and introduced into *Arabidopsis* and the hairy roots of soybean to further determine promoter activity. Histochemical analysis in transgenic *Arabidopsis* showed that GUS activity was mainly detected in roots and hypocotyls in all deletion fragments except GmPRP2p-471 (a 5’-deletion fragment of GmPRP2p-1062 with 471 bp length). GUS activity was higher in transgenic *Arabidopsis* and hairy roots with GmPRP2p-1062 and GmPRP2p-852 (a 5’-deletion fragment of GmPRP2p-1062 with 852 bp length) constructs than the other two constructs. GUS activity was enhanced by NaCl, PEG, IAA and JM treatments and decreased by SA, ABA and GA treatments in transgenic *Arabidopsis*.

**Conclusions:**

GmPRP2p-1062 is a root-preferential promoter, and its core fragment for root-preferential expression might lie between −369 and +1. GmPRP2p-852 may be useful in the genetic engineering of novel soybean cultivars in the future.

**Electronic supplementary material:**

The online version of this article (doi:10.1186/s12870-014-0245-z) contains supplementary material, which is available to authorized users.

## Background

Promoters play a very important role in the initiation and regulation of gene transcription, and they are important components in transgenic engineering [1]. Promoters can be divided into three types: constitutive, specific, and inducible. Constitutive promoters are widely used in genetic engineering. The cauliflower mosaic virus (CaMV) 35S promoter directs the expression of target genes in almost all tissues at all developmental stages [2]. However, the constitutive expression of transgenes is not always desirable for research and application. Constitutive expression for research purposes would conceal the elaborate function of transgenes, especially in signal transduction, energy transformation, and material transportation. Constitutive expression for applied purposes may cause an extra metabolic burden or toxic effects in transgenic plants. The repetitive use of the same promoter in genetic transformation is one of the major reasons for transgenic silencing [3-5]. Therefore, the development of specific or inducible promoters is necessary.

Inducible promoters are often regulated by particular chemical and physical factors, such as light, wounding, temperature, pH, hormones [6]. They can strongly enhance gene expression, and some of them are also specific [7].

Tissue-specific promoters are a type of specific promoters that drive gene expression within specific tissues or organs. Many specific promoters with different tissue specificities have been isolated and studied in transgenic research, including root-specific promoters [8-11], flower organ-specific promoters [12-15], seed-specific promoters [16,17], and fruit-specific promoters [18-20]. Hormones or abiotic stresses may regulate tissue-specific promoters. For example, wounds induce the rice *MT* promoter, which is preferentially expressed in roots and flowers [21]. The root-specific promoter, *PsPR10*, expresses higher GUS in response to the abiotic stresses NaCl, PEG6000 and mannitol as well as in response to SA, ABA and JA [9]. However, some tissue-specific promoters may have low activity or specificity, especially when these promoters drive gene expression in heterologous plants.

Soybeans are faced with severe root-related pathogens, pests, malnutrition and other abiotic stresses [22]. Soybean production suffers heavy losses from root-related biotic or abiotic stresses annually. For example, the soybean cyst nematode (*Heterodera glycines* Ichinohe) (SCN) was the primary cause for the suppression of soybean yield in the US from 2003 to 2005, and the yield suppression due to SCN in the US was approximately 8.3 million tons during these 3 years [23]. However, no efficient and economical methods have been developed to combat these diseases. Therefore, transgenic technology using root-specific promoters is promising because a transgenic soybean with a constitutive promoter has been successful worldwide [24].

Alfalfa *A9*, rice *Rcc2* and *Rcc3*, carrot *PRP1*, maize *ZRP3* and bean *PVR5*, which all encode a proline-rich protein (PRP), are expressed preferentially in root [25-28]. Three members of the *PRP* family are expressed in soybean with distinct, individual patterns of expression in different organs and at different development stages [29-31]. *GmPRP1* and *GmPRP2* exhibit root-specific expression [30,31]. Soybean *PRP1* mRNA is highly abundant in the elongating and mature region of the hypocotyls epidermal cells of seedlings. Soybean *PRP2* mRNA accumulates in phloem cells, and *PRP3* mRNA is specifically localized to the endodermoid layer of cells in the hypocotyl-elongating region [32,33]. *PRPs* are expressed with spatiotemporal specificity [34,35]. Moreover, factors associated with biotic and abiotic stresses also influence the expression of *PRPs* [36-39].

Some genes encoding PRPs have been isolated from soybean, but the function of the *PRP* promoters is not well characterized. Here, we cloned the *GmPRP2* promoter and studied its expression activity in transgenic *Arabidopsis* and soybean hairy roots.

## Results

### Detection of *GmPRP2* expression by quantitative realtime PCR

**Figure 1:**
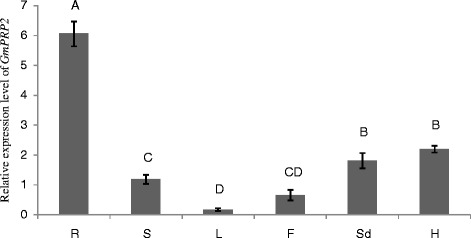
**The expression level of GmPRP2 by qRT-PCR in different tissues.** The relative expression level of GmPRP2 from real-time PCR was in different tissues. R, root; S, stem; L, leaf; F, flower; Sd, seed; H, hypocotyl. Data are the means of three replicates with SE shown by vertical bars. The capitals differ significantly by one-sided paired *t* test at P < 0.01.

*GmPRP2* expression was investigated by real-time PCR from root, stem, leaf, flower, seed and hypocotyl. The expression level was highest in the root, and second in the hypocotyl, seed and stem. The level in the leaf and flower were much lower than in the root (Figure 1). Therefore, *GmPRP2* gene showed a root-preferential expression.

### Cloning and sequence analysis of the *GmPRP2* promoter (GmPRP2p-1062)

**Figure 2:**
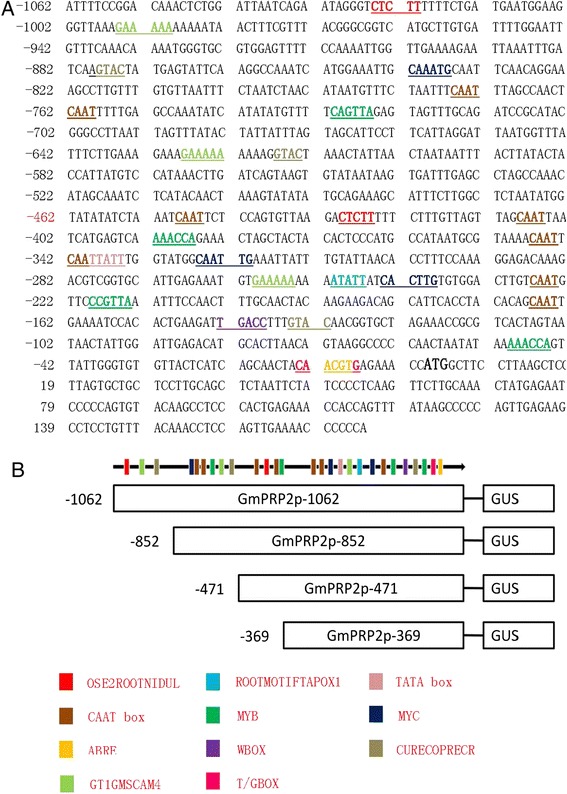
**Sequence of the GmPRP2p-1062 denoting the** ***cis*** **-elements predicted by the PLACE and PlantCARE databases and schematic diagrams of truncated GmPRP2p-1062 constructs. A** A 1,236 bp 5’-flanking region fragment of the *PRP2* gene, containing 1062 bp promoter sequence. The translated start site is defined as +1. The TATA box, partial CAAT box, Wbox, ABRE, MYB, MYC, root-specific elements are underlined with different colors. **B** Schematic diagrams of truncated GmPRP2p-1062 constructs. The numbers on the left indicate 5’-deletion fragments of the promoter. Some *cis*-elements are also marked with the colored columns.

A 1,236 bp 5’ flanking region fragment of the *PRP2* gene was amplified using first-round PCR. This fragment contained a 174 bp *GmPRP2* gene partial coding sequence. The second PCR product contained a 1,062 bp flanking sequence upstream of the translated initiation codon. The first nucleotide of *GmPRP2* cDNA was designated as +1 to orient the sequence numbers. The promoter sequence was analyzed using the PLACE and PlantCARE web tools. Several putative *cis*-regulatory elements were deciphered from the promoter sequence of *GmPRP2* (Figure 2A). TATA box sequence elements, which were required for critical and precise transcription initiation, were found in the −317 region of the sequence. CAAT BOX sequences, which were responsible for the tissue-specific promoter activity, were found at numerous positions: −776, −762, −449, −409, −347, −342, −227, and −167. OSE2ROOTNODULE sequences, which were responsible for an organ-specific promoter activity in infected cells of root nodules, were found at −1000 and −430. ROOTMOTIFTAPOX1 sequence elements, which were required for organ specificity, were identified at the −251 position. OSE2ROOTNODULE elements and ROOTMOTIFTAPOX1 element are critical for root-specific expression.

Other important promoter elements and their putative functions are photographed (Figure 2A) and listed (Additional file 1). The ABRELATERD1 site at −12 and ACGTATERD1 site at −282 and −12 were responsible for dehydration. ARR1AT transcription factors for genes were located at −1044, −955, −616, −542, −147, and −93. The copper- and oxygen-responsive element, CURECORECR, was found at the −878, −617, and −135 positions. GT1GMSCAM4 participated in pathogen- and salt- induced SCaM-4 gene expressions and presented at −995, −628, and −260. MYB1AT and MYBCORE binding sites of MYB were found at −392, −50, −731, and −219. Hormone-responsive elements, T/GBOXATPIN2, involved in jasmonate signaling and WBOXNTERF3, involved in the activation of the ERF3 gene, were sited at −13 and −143, respectively. Some light-responsive transcription elements, such as EBOXBNNAPA (−842, −326, −244, −167), GT1CONSENSUS (−995, −850, −628, −260, −162) and INRNTPSADB (−778, −764), were also found in the promoter region.

Serial 5’-deletion fragments were created to identify the core region of the GmPRP2p-1062 that controlled tissue-specific expression: GmPRP2p-852, GmPRP2p-471 and GmPRP2p-369, which were 852, 471 and 369 bp in length, respectively, according to the density of the *cis*-elements on the promoter sequence (Figure 2B).

### Spatiotemporal expression patterns of GmPRP2p-1062 and the 5’-deletion fragments in *Arabidopsis*

**Figure 3:**
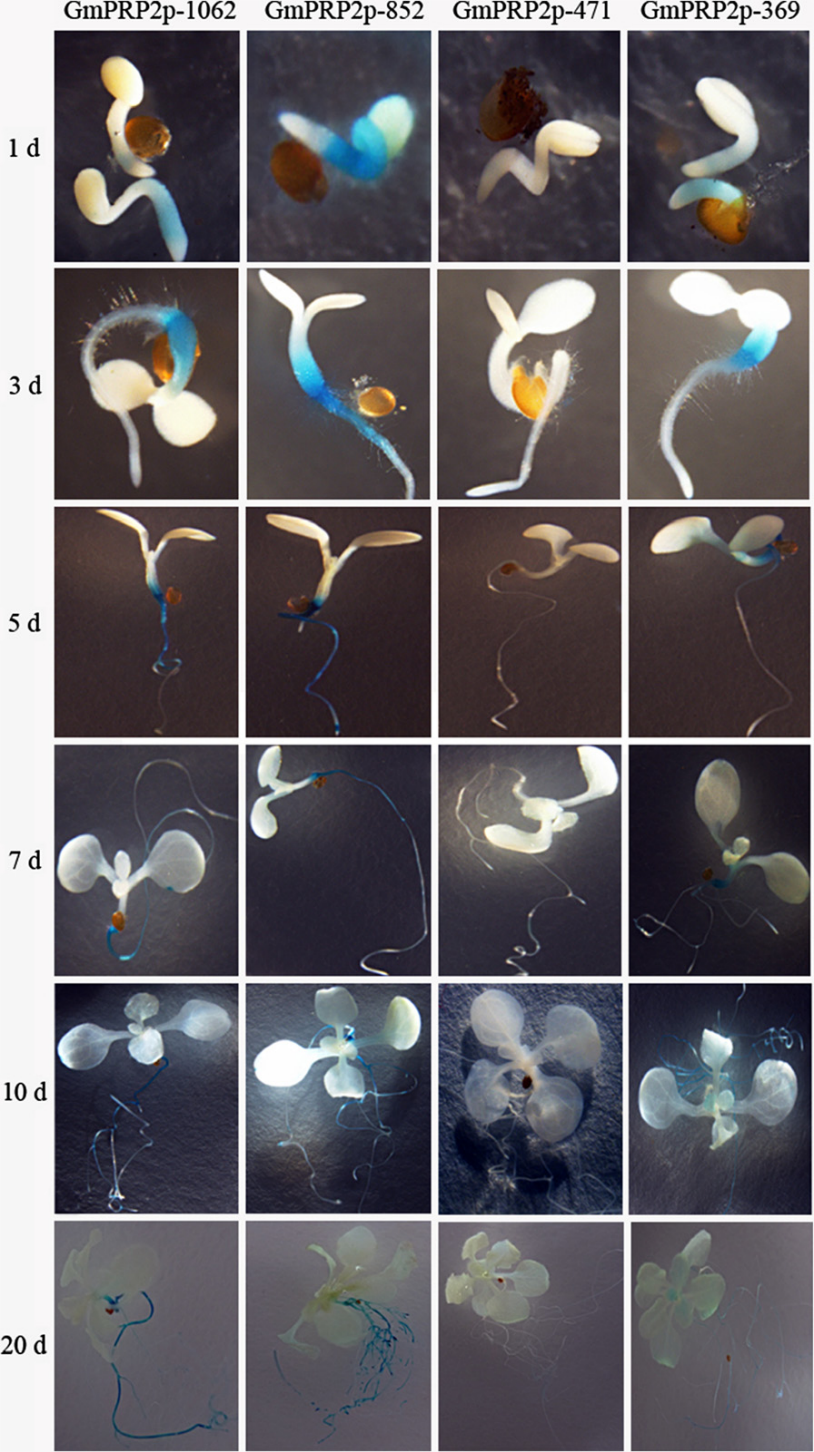
**GUS histochemical assays in T**_**3**_ **transgenic** ***Arabidopsis*** **seedlings carrying GmPRP2p-1062, GmPRP2p-852, GmPRP2p-471 and GmPRP2p-369 constructs.** Photographs were taken 1 d, 3 d, 5 d, 7 d, 10 d, and 20 d from seeds placed from 4°C to 22°C on 1/2 MS plates.

GmPRP2p-1062 driven GUS expression was monitored during plant development and in various organs using histochemical staining in T_3_ transgenic lines of *Arabidopsis* grown on 1/2 MS medium to precisely define the spatiotemporal expression pattern of the *GmPRP2* promoter. GUS expression was detected in hypocotyls and roots in 1-day-old and 3-day-old seedlings carrying the GmPRP2p-1062 construct, but GUS staining was not detected in the cotyledons and apical roots (Figure 3). GUS expression was detected strongly in roots of the 5-day-old and 7-day-old seedlings, but the staining was a little weak in the hypocotyls. GUS expression in 10-day-old and 20-day-old seedlings was similar to that of the younger seedlings.

**Figure 4:**
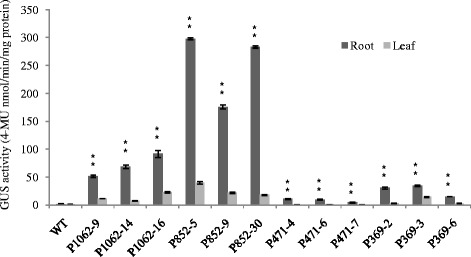
**GUS activity assays in T**_**3**_ **transgenic** ***Arabidopsis*** **roots and leaves carrying GmPRP2p-1062, GmPRP2p-852, GmPRP2p-471 and GmPRP2p-369 constructs.** GUS activity assays in T_3_ transgenic *Arabidopsis* roots and leaves carrying different deletion fragments of the GmPRP2p-1062. Each promoter fragment had three transgenic lines. P1062-9, P1062-14, and P1062-16 are the three transgenic lines carrying the GmPRP2p-1062 construct; P852-5, P852-9, and P852-30 are the three transgenic lines carrying the GmPRP2p-852 construct; P471-4, P471-6, and P471-7 are the three transgenic lines carrying the GmPRP2p-471 construct; P369-2, P369-3, and P369-6 are the three transgenic lines carrying the GmPRP2p-369 construct. Data are the means of three replicates with SE shown by vertical bars. **differ significantly by one-sided paired *t* test at P < 0.01.

The 5’-deletion fragments were also fused to the GUS gene and transformed into *Arabidopsis*. GUS staining showed that the GmPRP2p-852 and GmPRP2p-369 constructs presented similar expression patterns as the GmPRP2p-1062 construct, which was high in roots and hypocotyls, except leaves. GUS staining was not visible with the GmPRP2p-471 construct (Figure 3). Fluorometric GUS assays revealed that GmPRP2p-1062 and GmPRP2p-852 constructs drove strong GUS activity in roots, and GUS activity was significantly reduced in GmPRP2p-471 and GmPRP2p-369 constructs compared with GmPRP2p-1062 and GmPRP2p-852 constructs (Figure 4). The 5’-deletions of the GmPRP2p-1062 from −1062 to −852 slightly increased GUS activity. A further deletion to −471 obviously decreased GUS activity, and a deletion to −369 was slightly better than −471. However, the GUS expression level in these plants was much lower than in the transgenic plants with GmPRP2p-1062 and GmPRP2p-852 constructs. We speculated that the promoter fragment from −471 to −369 may contain the suppressor which induced the expression level increased in GmPRP2p-369 compared with GmPRP2p-471. The GUS activity in roots was obviously higher than in leaves, regardless of the promoter fragment except GmPRP2-471. The results of GUS staining and fluorometric GUS assays were uniform.

**Figure 5:**
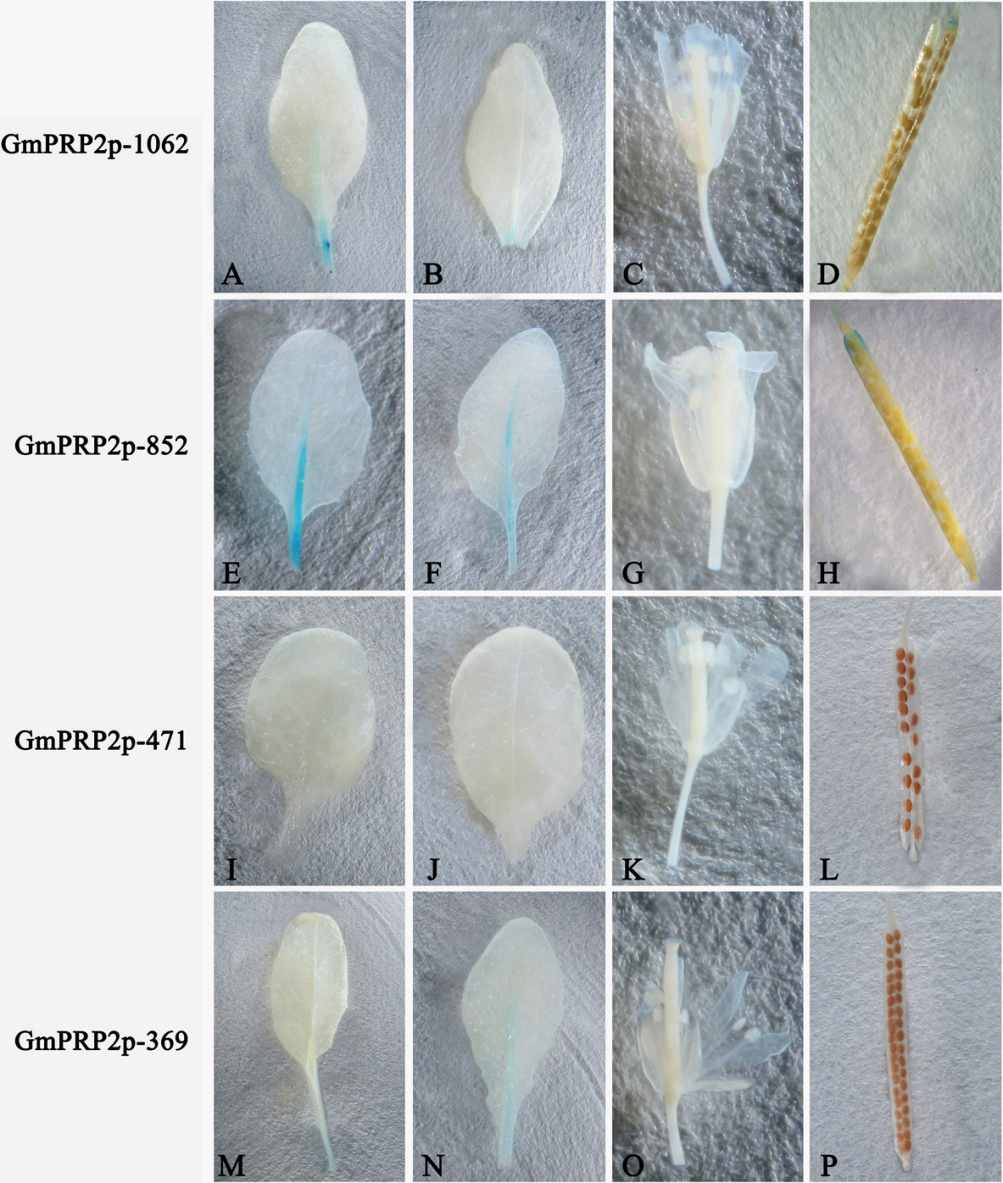
**GUS histochemical assays in T**_**3**_ **transgenic** ***Arabidopsis*** **seedlings carrying GmPRP2p-1062, GmPRP2p-852, GmPRP2p-471 and GmPRP2p-369 constructs in the reproductive stage. A**, **E**, **I**, **M** Leaves of 30-day-old seedlings of transgenic plant grown on soil with GmPRP2p-1062, GmPRP2p-852, GmPRP2p-471, and GmPRP2p-369 constructs, respectively. **B**, **F**, **J**, **N** Leaves of 40-day-old seedlings of transgenic plant grown on soil with GmPRP2p-1062, GmPRP2p-852, GmPRP2p-471, and GmPRP2p-369 constructs, respectively. **C**, **G**, **K**, **O** Floral organs of transgenic plants with GmPRP2p-1062, GmPRP2p-852, GmPRP2p-471, and GmPRP2p-369 constructs, respectively. **D**, **H**, **L**, **P** Silique of transgenic plants with GmPRP2p-1062, GmPRP2p-852, GmPRP2p-471, and GmPRP2p-369 constructs, respectively.

GUS expression was observed in the roots of all promoter fragment constructs, except the GmPRP2p-471 construct. GUS staining appeared in the petiole during the reproductive growth stage, and emerged in the split margins besides the abscission in siliques, but the expression levels were quite weak. GUS staining was not detected in flowers and seeds. Furthermore, GUS staining was not visible in any organs of plants containing the GmPRP2p-471 construct (Figure 5).

### MeJA and IAA increased and GA, ABA and SA decreased the GUS activity of the GmPRP2p-1062 and 5’-deletion fragments

**Figure 6:**
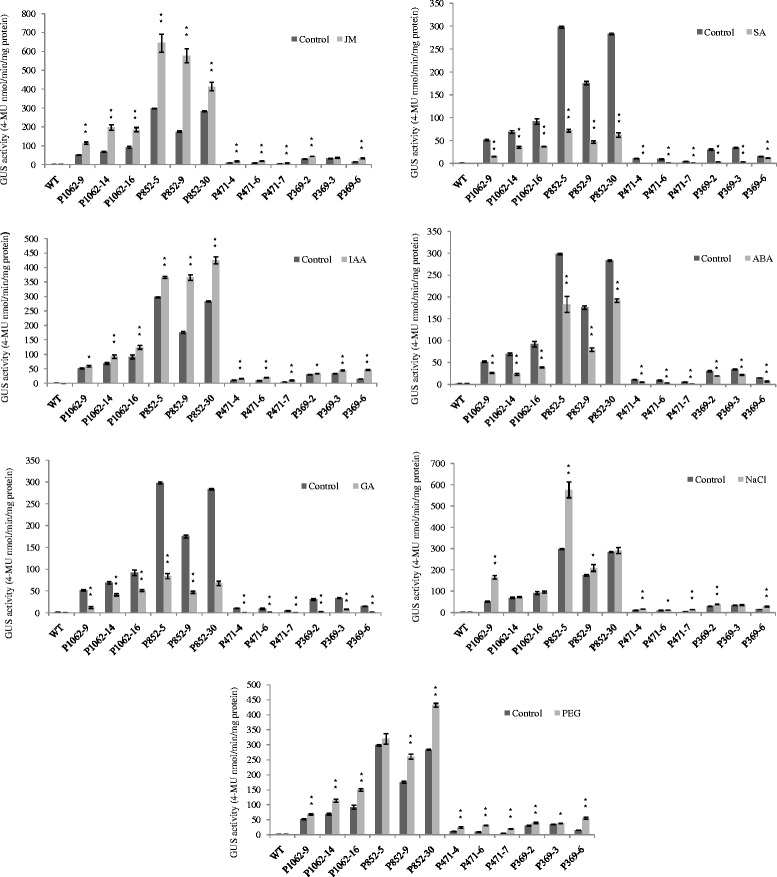
**GUS activity of transgenic** ***Arabidopsis*** **with different constructs (GmPRP2p-1062, GmPRP2p-852, GmPRP2p-471, GmPRP2p-369) in roots in responses to MeJA, IAA, SA, GA, ABA, NaCl, PEG.** The GUS activity for each treatment was measured in 20-day-old seedlings from each of three independent transgenic lines. Transgenic seedlings were treated for 24 h. Control seedlings were treated with water. P1062-9, P1062-14, and P1062-16 are the three transgenic lines carrying the GmPRP2p-1062 construct; P852-5, P852-9, and P852-30 are the three transgenic lines carrying the GmPRP2p-852 construct; P471-4, P471-6, and P471-7 are the three transgenic lines carrying the GmPRP2p-471 construct; P369-2, P369-3, and P369-6 are the three transgenic lines carrying the GmPRP2p-369 construct. Data are presented as the means of three replicates with SE shown by vertical bars. *and** differ significantly by one-sided paired *t* test at P < 0.05 and P < 0.01, respectively.

We tested the GUS activity in the roots of 20-day-old transgenic seedlings treated with 1 mM SA, 100 μM MeJA, 100 μM IAA, 100 μM GA, and 100 μM ABA for 24 h to assess the response of the GmPRP2p-1062 and 5’-deletion fragments to various hormones. Seedlings treated with H_2_O and wild type seedlings treated with the above stressors were used as controls. MeJA enhanced the GUS activity in transgenic seedlings containing the GmPRP2p-1062 construct compared to H_2_O control samples (Figure 6). The GUS activity was increased by 2.22-fold (P1062-9), 2.86-fold (P1062-14) and 2.02-fold (P1062-16) in the three transgenic lines. Similar results were observed in seedlings transformed with the GmPRP2p-852, GmPRP2p-471 and GmPRP2p-369 constructs. The GUS activity was increased one to three times in transgenic lines. IAA increased the GUS activity in most transgenic seedlings with different constructs by one to two times (Figure 6).

SA and GA treatments decreased the GUS activity approximately 40-70% in transgenic seedlings with the GmPRP2p-1062 construct and approximately 70-80% in transgenic seedlings with the GmPRP2p-852 construct. The GUS activity was decreased 70-90% in transgenic seedlings with the GmPRP2p-471 and GmPRP2p-369 constructs. ABA treatment slightly decreased the GUS activity approximately 30-60% in most transgenic seedlings compared to SA and GA treatments (Figure 6).

### NaCl and PEG increased the activity of the GmPRP2p-1062 and the 5’-deletion fragments

We tested GUS activity in the roots of 20-day-old transgenic seedlings treated with 200 mM NaCl and 20% PEG6000 for 24 h to identify the response of GmPRP2p-1062 and 5’-deletion fragments to environmental stresses. The seedlings treated with H_2_O and wild type seedlings treated with the above stressor were used as controls. NaCl and PEG treatment enhanced GUS activity. NaCl treatment increased GUS activity by 3.19-fold (P1062-9), 1.05-fold (P1062-14) and 1.05-fold (P1062-16) in the three GmPRP2p-1062 transgenic lines. The GUS activity was increased one to two times in most of the transgenic lines with the other constructs (Figure 6). PEG treatment increased the GUS activity in all transgenic lines carrying different constructs, and greatly increased the GUS activity by 2.29-fold (P471-4), 3.24-fold (P471-6) and 4.08-fold (P471-7) in the GmPRP2p-471 transgenic lines (Figure 6). However, the GUS levels were much lower in the GmPRP2p-471 transgenic lines than in the GmPRP2p-1062 and GmPRP2p-852 transgenic lines (Figure 6).

### The expression patterns of GmPRP2p-1062 and the 5’-deletion fragments in soybean hairy roots

**Figure 7:**
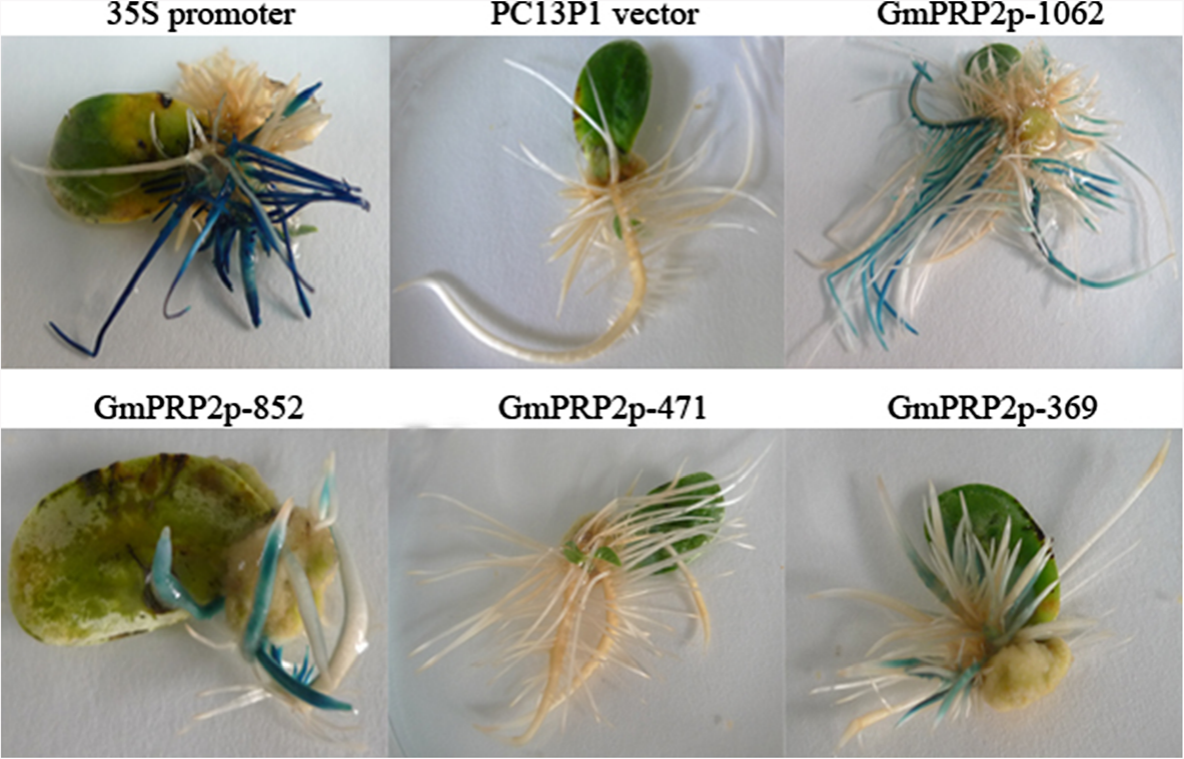
**GUS histochemical assays in transgenic soybean hairy roots.** GUS staining of the transgenic cotyledon with hairy roots carrying 35S promoter construct, PC3P1 vector, GmPRP2p-1062 construct, GmPRP2p-852 construct, GmPRP2p-471 construct, and GmPRP2p-369 construct.

Numerous branched roots developed from each wound site on the cotyledon after *Agrobacterium rhizogenes* K599 infection and co-cultivation. The entire cotyledon with hairy roots was used for GUS staining. The 35S promoter drove strong GUS staining (Figure 7). GmPRP2p-1062, GmPRP2p-852 and GmPRP2p-369 also drove GUS staining in soybean hairy roots, but GUS staining was not detected in transgenic GmPRP2p-471 hairy roots. The transformant PC13P1 vector was used as a negative control.

**Figure 8:**
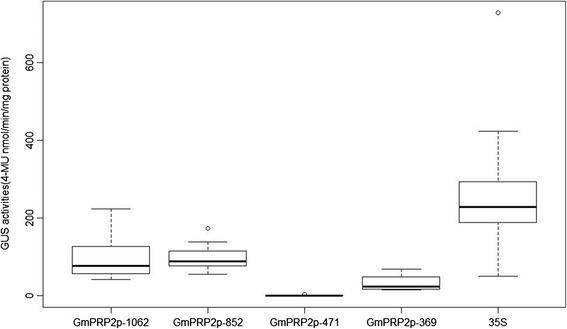
**GUS activity of transgenic soybean hairy roots with different constructs (GmPRP2p-1062, GmPRP2p-852, GmPRP2p-471, GmPRP2p-369, 35S).** Box plot analysis of GUS activities obtained in transgenic soybean hairy roots with different constructs (GmPRP2-1062, GmPRP2-852, GmPRP2-471, GmPRP2-369, 35S). GUS activity is given in nmol 4-MU min^−1^ mg^−1^ total protein. The lower boundary of each box denotes the 25^th^ percentile for each promoter, the solid line within each box denotes the 50^th^ percentile, the upper boundary of each box denotes the 75^th^ percentile. Two ends of the vertical line denote the maximum and minimum. Outlying data are indicated by empty black circles.

We used GUS staining to select positive roots because the hairy roots from cotyledon were not all positive. For each promoter construct, twenty soybean cotyledons as explants were used to transform. Thirty to fifty hairy roots from each transgenic promoter cotyledons were examined for GUS staining. The hairy roots of transgenic GmPRP2p-471 constructs were also detected using PCR. Ten GUS-positive hairy roots of each transgenic construct were used to measure GUS activity. The GUS activity results were used for statistical analysis. The 35S promoter drove the highest GUS expression level compared to the maximum expression level of each transgenic construct (Figure 8). The GUS expression levels in all transgenic GmPRP2p-1062 and 5’-deletion fragments were lower than that in the 50^th^ percentile of the 35S promoter. The GUS expression levels for GmPRP2p-1062 and GmPRP2p-852 were higher than those of the lowest GUS expression level of the 35S promoter. The GUS expression levels of most hairy roots for GmPRP2p-369 were less than the minimum of the 35S promoter, GmPRP2p-1062 and GmPRP2p-852. The expression activity of GmPRP2p-471 was extremely weak, and GUS staining was not detectable by eyes. The maximum GUS expression of GmPRP2p-1062 was higher than that of GmPRP2p-852. But the GUS expression in the 50^th^ percentile of GmPRP2p-1062 lines was lower than those of GmPRP2p-852 (Figure 8).

## Discussion

PRPs are a structural cell wall protein in plants [37]. Previous studies of PRPs predominantly focused on gene expression and showed that PRPs are regulated spatiotemporally during the development of a particular tissue or cell type [29-31]. For example, *Arabidopsis PRP1* and *PRP3* were exclusively expressed in roots, and *PRP2* and *PRP4* transcripts were abundant in aerial organs of the plant [40,41]. The rice *OsPRP3* expression was mainly present in flowers and accumulated during the late stage of flower development [42]. *OsPRP1* is expressed preferentially in spikelets and buds, but expression is lower in roots and leaves [43]. The cotton *GhHyPRP3* mRNA was abundant in petals and 10-DPA ovules but lower in roots and cotyledons and absent in leaves and anthers [44]. Therefore, PRPs exhibit an organ-preferential expression pattern to meet the functional and physical requirements of different cell types at different developmental stages.

PRP promoters play an important role in the organ-preferential expression pattern of PRPs. Xu *et al*. reported that *GhPRP5* is a fiber-specific gene, and its promoter directs *GUS* expression only in the trichomes of both transgenic *Arabidopsis* and tobacco plants [32]. The transcript of the alfalfa *MsPRP2* gene is expressed in a root-specific manner, and *GFP* driven by the *MsPRP2* promoter is also expressed in the root [45]. These studies promote an understanding of the mechanism of organ-preferential expression and provide more promoter options for genetic engineering.

The expression of *GmPRP1* and *GmPRP2* in soybeans is also root-specific [29-31], but little is known about their promoters and regulatory mechanisms. Here, we cloned the *GmPRP2* promoter (GmPRP2p-1062), which contained many important *cis*-elements, such as the TATAbox, CAATbox, MYB, MYC, ABRE and root-related elements (Figure 2).

GUS staining showed a root-preferential expression in the vegetative stage in the model plant *Arabidopsis* (Figure 3). Therefore, GmPRP2p-1062 can harbor some *cis*-regulatory elements and drive a root-preferential expression. Three 5’-deletion fragments (GmPRP2p-852, GmPRP2p-471, GmPRP2p-369) also exhibited root-preferential expression, but the expression levels were significantly different. In our study, we detected that expression level in GmPRP2p-471 was very low, then when a further deletion the expression level in GmPRP2p-369 was increased, so we speculated that the suppressor may exist in the promoter fragment from −471 to −369. The elements in these deletion regions, which might be responsible for the root specificity and expression, require further research. The study of GmPRP2p-1062 and 5’-deletion fragments in soybean hairy roots showed that the expression patterns were similar to the transgenic *Arabidopsis* (Figure 7), and GUS activity was much higher with the GmPRP2p-1062 and GmPRP2p-852 constructs than the other two constructs. The homogeneity in soybean and *Arabidopsis* inferred that the *PRP2* promoter has a similar expression pattern in dicotyledonous plants. The expression level was highest in transgenic *Arabidopsis* with the GmPRP2p-852 construct, and the range of GUS activity varied little between the ten transgenic hairy roots with the GmPRP2p-852 construct compared with the other constructs (Figure 8). Therefore, the GmPRP2p-852 may be suitable for application.

Histochemical staining of GUS activity during reproductive growth was also observed in the petiole of green leaves and the split margins in siliques in all promoter constructs except the GmPRP2p-471, but the expression level was much lower than in roots. GUS staining was not observed in flowers and seeds (Figure 5). GUS staining was also detected in the main veins in transgenic lines with the GmPRP2P-852 construct, which exhibited high expression levels. This result indicated that *GmPRP2* may be expressed in only a few tissues during the reproductive stage and suggested that the promoter may be suitable for genetic engineering with little concern about food safety.

Biotic and abiotic stresses also influenced the expression of *PRPs*. Some evidence indicated that many internal and external factors, such as wounding, fungal infection, circadian rhythm, salt stress, drought stress, and plant regulators, up or down regulates the expression of *PRPs* [36,38]. For example, Alfalfa *MsPRP2* is salt-inducible [46]. Low temperature induced transcripts of *Brassica napus BnPRP*. The bean *PvPRP1* mRNA initially decreases then increases in wounded hypocotyls and decreases in the elicitor [38]. Cotton *GhHyPRP3* transcription in roots was up-regulated by salt stress, cold stress, and osmotic stress and down-regulated by GA_3_ [44]. *PRP* gene expression is complicated with positive or negative regulation by stresses. The diversity of the promoter may regulate gene expression in many biological processes. Our results showed that the activity of the GmPRP2p-1062 promoter was differentially regulated in response to various abiotic factors. NaCl, PEG, JM and IAA up-regulated the activity, and ABA, GA, and SA down-regulated the activity (Figure 6). The deletion fragments contained stress elements. The −1062 to −853 fragment contained a copper- and salt-responsive element, and −852 to −472 contained light- and salt-responsive elements. The three 5’-deletion fragments showed a similar response to the stress factors treated with the GmPRP2p-1062. The region from −369 to +1 is rich in various biotic and abiotic stress-related *cis-*elements, including four CAATBOX, two MYB and three MYC *cis*-elements, two ARR1 *cis*-elements, one ABRE *cis*-element, one WBOX and one T/GBOX (Additional file 1), and this region is a crucial part of the promoter. MYB and MYC recognition sites may contribute to the activation of drought- and ABA-regulated gene expression. ABRE was identified as an ABA-responsive element. WBOX was involved in the activation of the ERF3 gene by wounding, and the T/GBOXATPIN2 element at −13 was involved in jasmonate signaling (Additional file 1). The above studies demonstrated that the *PRP2* promoter may regulate gene expression on the transcriptional level.

The fine regulation of transgene expression levels is not easy to obtain due to the lack of available promoters. This work provided important insights into the promoter regions that control spatial- and stress-specific expression. This promoter could be widely used as a tool in genetic engineering. Soybeans are an important source of nutrition worldwide, but soybeans are generally considered salt- and drought-sensitive. Soybean roots also suffer from severe diseases that lead to large annual losses. This promoter is promising for the engineering of resistance traits in soybeans.

## Conclusions

GmPRP2p-1062 is a root-preferential promoter, and the core fragment for root expression might reside between −369 and +1. The expression activity for this promoter was lower with shorter promoter sequences from −471 to −369 bp, and environmental stresses altered expression activity. GmPRP2p-852 may be used in soybean genetic engineering to improve root tolerance and resistance in the future.

## Methods

### Plant material and growth condition

Seeds of the soybean cultivar Williams 82 were grown in pots filled with vermiculite under 12 h light/12 h dark cycles at 28°C. The root tissues were cut for genomic DNA extraction after the first true leaf opening. Seeds of *Arabidopsis thaliana* (Columbia) were surface-sterilized with 10% NaClO for 15 min and washed four times with sterile water. The sterilized *Arabidopsis* seeds were placed on 1/2 MS medium, and the plates were transferred to a plant growth incubator for seed germination under 16 h light/ 8 h dark at 22°C after stratification at 4°C for 3 days.

### Analysis of GmPRP2 tissue expression pattern by qRT-PCR

The total RNA was isolated from soybean different tissues of root, stem, leaf, flower, seed, and hypocotyl using Trizol agent (Trans). cDNAs were synthesized from 800 ng total RNA using the M-MLV reverse transcriptase and oligodT primer according to the manufacturer’s instructions (Promega). qRT-PCR reactions (20 μl volume containing 2 μl cDNA as the template) were performed using the StepOne real-time PCR system (Applied Biosystems 7500) in standard mode with the KAPA SYBR FAST Universal qRT- PCR kit (KAPA Biosystems). Each tissue has three samples, and each sample of one tissue was performed in triplicate. The soybean *Actin* gene was used as the internal control. The primers for *GmPRP2* were 5′- GCTCCTTAGTGCTGCTCCTT-3′ and 5′-TCAGTGGGAGGCTTGTACA-3′. The primers for *Actin* were 5′-CGTTTCATGAATTCCAGTAGC-3′ and 5′- GAGCTATGAATTGCCTGATGG-3′.

### Cloning of the *GmPRP2* promoter

Genomic DNA was isolated from the roots of soybean using the CTAB method. Two primers were designed according to the sequence of *GmPRP2* and its upstream sequence from the soybean genome database: the upstream primer F1 (5′-ATTTTCCGGACAAACTCTGG-3′) and downstream primer R1 (5′-TGGGGGGTTTTCAACTGGAG-3′). PCR cycling parameters were as follows: 94°C 5 min; 94°C 30 s, 55°C 30 s, 72°C 2 min, 35 cycles; 72°C for 10 min. The first PCR product contained a partial coding sequence, so another downstream primer R2 (5′-GGTTTCTCACGTTGTAGTTG-3′) that contained no coding sequence was designed. The second round of PCR was amplified with the F1 and R2 primers using the first PCR product as the template. The PCR cycling parameters were as follows: 94°C 5 min; 94°C 30 s, 52°C 30 s, 72°C 2 min, 35 cycles; 72°C for 10 min. The PCR products were cloned into pEASY-T1 vector and sequenced.

### PCR amplification of 5’-deletion fragments of the *GmPRP2* promoter

We designed three forward primers to obtain 5’-deletion fragments of the *GmPRP2* promoter: F-852 (5′-ATGGAAATTGCAAATG-3′), F-471 (5′-CTAATATGGTATATATC-3′), and F-369 (5′-TCCCATGCCATAATGCG-3′). The same reverse primer, R2, amplified the promoter deletion at −852 (F-852/R2), −471 (F-471/R2), and −369 (F-369/R2), respectively. The PCR cycling parameters were as follows: 94°C 5 min; 94°C 30 s, 52°C 30 s, 72°C 1 min, 35 cycles; 72°C for 10 min. The promoter fragments were cloned into pEASY-T1 vector and confirmed by sequencing. The *GmPRP2* promoter was named the GmPRP2p-1062, and the three 5’-deletion fragments were named GmPRP2p-852, GmPRP2p-471, and GmPRP2p-369.

### Bioinformatics analysis of the promoter sequence

Regulatory elements in promoter regions were analyzed using the online program PLACE (a database of plant *cis*-acting regulatory DNA elements) [47] and PlantCARE (a database of plant *cis*-acting regulatory elements, enhancers and repressors) [48]. These two programs are available at http://www.dna.affrc.go.jp/PLACE/ and http://bioformatics.psb.ugent.be/webtools/plantcare/html/, respectively.

### Construction of the promoter-GUS reporter plasmid and *Arabidopsis* transformation

*Sac*I and *Xba*I digested the pEASY-T1 vector (TransGene Bioteck) and pC13P1 vector (Additional file 2). A 1062 bp *GmPRP2* promoter was ligated into the vector pC13P1 with *Sac*I and *Xba*I digestion to construct the GmPRP2p::GUS vector. The 5’-deletion promoter fragments were fused to the GUS reporter gene of the pC13P1 vector using the same method described above.

The constructs and a selected vector pC(Delt)GUS (Additional file 3) plasmid were introduced into *Agrobacterium tumefaciens* GV3101 using electroporation [21]. The *Agrobacterium* liquids (1:1 v/v) mixed with the constructs and pC(Delt)GUS vector were transformed into *Arabidopsis* (Col-0) using the flower dipping method [49]. Transformants were selected by planting the seeds on 1/2 MS plates containing 50 mg/L hygromycin B. The positive transformants were confirmed using PCR. The PCR primers were F1/R2 for transgenic GmPRP2p-1062 plants, F-851/R2 for transgenic GmPRP2p-851 plants, F-471/R2 for transgenic GmPRP2p-471 plants, and F-369/R2 for transgenic GmPRP2p-369 plants. The homozygous transgenic plants were confirmed using genetic analysis of the segregation ratio of later generations.

### Treatment with abiotic stress

To characterize the induced activities of the GmPRP2p-1062 and 5’-deletion fragments in response to different defense signal molecules, we treated transgenic *Arabidopsis* plants with NaCl, PEG6000, abscisic acid (ABA), salicylic acid (SA), methyl jasmanate (MeJA), indole-3-acetic acid (IAA), and gibberellins acid (GA). Twenty-day-old transgenic plants were used for the following treatments. The roots were immersed in 200 mM NaCl, 20% PEG6000, 100 μMABA, 1 mM SA, 100 μM JM, and 100 μM GA water solutions for 24 h, frozen in liquid nitrogen and stored at −80°C for protein extraction and GUS fluorometric assays. The control plants were incubated in water. The wild type *Arabidopsis* also served as a control.

### Soybean hairy root transformation

The plasmids of GmPRP2p-1062 and 5’-deletion fragments vectors were introduced into the *Agrobacterium rhizogenes* strain K599 to induce expression in hairy roots [50]. Soybean cv. Zigongdongdou was screened for transformation. Seeds were surface sterilized for 16 h using chlorine gas, which was produced by mixing 3.5 mL of 12 N HCl and 100 mL commercial bleach in a tightly sealed dessicator. Sterilized seeds were germinated in B5 medium, and entire cotyledons from 5-day-old seedlings as explant were harvested and wounded with a scalpel, which was previously dipped into an overnight culture of *A. rhizogenes* strain K599 carrying GmPRP2p-1062::GUS vector, GmPRP2p-852::GUS vector, GmPRP2p-471::GUS vector, GmPRP2p-369::GUS vector, 35S::GUS vector and PC13P1 vector. Subsequently, the entire cotyledons were immersed into *A. rhizogenes* culture and shaken (50 rpm) at 25°Cfor 30 min. Explants were dried on sterile filter paper and transferred to co-cultivation medium (CCM) containing 10% MS, 3.9 g/L morpholino ethanesulfonic acid, 150 mg/L cysteine and 150 mg/L dithiothreitol. The CCM was covered with sterile filter paper and incubated under a 16 h light/8 h dark cycle condition at 24°C. Entire cotyledons were cultured 5 days later on 1/2 MSB medium (1/2 Murashige and Skoog basal nutrient salts, B5 vitamins, 3% Suc and 3 g/L phytagel, pH 5.7) [50]. Approximately 10–12 d after root emergence, 1 cm-long root tips or the bottoms of each root were cut for GUS staining. We selected GUS-positive roots for measurement of GUS activity.

### GUS histochemical and fluorometric analyses

Histochemical and fluorometric GUS assays were performed according to Jefferson [51]. The tissues were placed in GUS staining solution (50 mM sodium phosphate, pH7.0, 0.5 mM potassium ferrocyanide, 0.5 mM potassium ferricyanide, 0.5 mg/ml 5-bromo-4 chloro-3-indolyl-β-D- glucuronide (X-Gluc), 0.1% Triton X-100 and 20% methanol) and incubated at 37°C overnight. After staining, tissue samples were bleached with 50% ethanol, 70% ethanol and 90% ethanol for 1 h each and immersed in 70% ethanol overnight. GUS staining was observed under a Nikon SMZ1500 microscope and photographed with a Nikon DS-Fil.

Tissues were homogenized to a fine power with liquid nitrogen and vortexed with 1 ml GUS extraction buffer (50 mM sodium phosphate, pH 7.0, 10 mM EDTA, pH 8.0, 10 mM β-mercaptoethanol, 0.1% Triton X-100) for the fluorometric GUS assays. The samples were centrifuged for 10 min at 15,000 rpm, 4°C, and the supernatant was collected. Each sample was incubated in assay buffer (4-methylumbelliferyl-β-D-glucuronide (4-MUG)) at 37°C for 60 min. The reaction was stopped by the addition of 0.2 M Na_2_CO_3_. One blank was prepared per sample with a 0-min incubation. Fluorescence was measured on an F-280 Luminescence Spectrometer with excitation at 365 nm and emission at 455 nm. Protein concentration in supernatant was assessed using the Bradford method with bovine serum albumin (BSA) as a standard [52]. GUS activity was calculated as nanomoles of 4-Methylumbelliferone (4-MU) per minute per milligram of protein.

## Additional files

Additional file 1: Table S1. Putative *cis*-acting elements in the GmPRP2p-1062 by PLACE and PlantCARE. Additional file 2: Figure S1. The pC13P1 vector. Additional file 3: Figure S2. The pC(Delt)GUS vector.

## Abbreviations

PRP: Proline-rich protein
GUS: β-Glucuronidase gene
MS: Murashige and Skoog medium
IAA: Indole-3-acetic acid
MeJA: Methyl jasmanate
ABA: Abscisic acid
SA: Salicylic acid
GA: Gibberellins acid
MU: 4-Methylumbelliferone
BSA: Bovine serum albumin
MUG: 4-methylumbelliferyl-β-D-glucuronide
X-Gluc: 5-bromo-4 chloro-3-indolyl-β-D- glucuronide
