# Supplementary material for: GmPRP2 promoter drives root-preferential expression in transgenic Arabidopsis and soybean hairy roots
Source: BMC Plant Biol. 2014 Sep 16;14:245. doi: 10.1186/s12870-014-0245-z (PMC4172956; doi:10.1186/s12870-014-0245-z)
Supplement: Additional file 1: Table S1. — Putative cis-acting elements in the GmPRP2p-1062 by PLACE and PlantCARE. [file 12870_2014_245_MOESM1_ESM.docx]

**Table 1 Putative *cis*-acting elements in the GmPRP2p-1062 by PLACE and PlantCARE**

| Name of *cis*-element | Sequence | Position from translation start site | Function |
| --- | --- | --- | --- |
| ABRELATERD1  ACGTATERD1 | ACGTG  ACGT | -12  -282 -12 | Early responsive to dehydration  Induction by dehydration stress and dark-induced senescence |
| ARR1AT | NGATT | -1044 -955-616 -542 -147 -93 | Transcriptional activators |
| CAATBOX1 | CAAT | -776 -762 -449 -409 -347 -342 -227 -167 | Sequences responsible for the tissue specific promoter activity |
| CURECORECR | GTAC | -878 -617 -135 | Copper- and oxygen–responsive elements |
| EBOXBNNAPA | CANNTG | -842 -326 -244 -167 | *Cis*-element binding BHLH factor involved in light responsiveness |
| GT1CONSENSUS | GRWAAW | -995 -850 -628 -260 -162 | GT-1 binding site in many light-regulated genes |
| GCN4OSGLUB1 | TGAGTCA | -399 | Identification of *cis*-regulatory elements required for endosperm expression |
| GT1GMSCAM4 | GAAAAA | -995 -628 -260 | Plays a role in pathogen- and salt-inducedSCaM-4 gene expression |
| INRNTPSADB | YTCANTYY | -764 -778 | Light-responsive transcription |
| MYB1AT | WAACCA | -392 -50 | MYB recognition site |
| MYBCORE | CNGTTR | -731 -219 | Binding site for MYB and responsive to water stresses |
| MYCCONSENSUSAT | CANNTG | -842 -326 -244 -167 | MYC recognition site |
| OSE2ROOTNODULE | CTCTT | -1000 -430 | Organ-specific elements in infected cells of root nodules |
| POLLEN1LELAT52 | AGAAA | -996 -633 -629 -569 -387 -268 -122 -7 | Responsible for pollen specific activation |
| ROOTMOTIFTAPOX1 | ATATT | -251 | The root specificity element |
| TATA BOX | TATA | -317 | Essential for promoter recognition |
| T/GBOXATPIN2 | AACGTG | -13 | Involved in jasmonate signaling |
| WBOXNTERF3 | TGACY | -143 | Involved in activation ofERF3 gene by wounding |

N indicates A, C, G or T; W indicates A or T; Y indicates C or T; R indicates A or G.
