# Supplementary figures and images for: GmPRP2 promoter drives root-preferential expression in transgenic Arabidopsis and soybean hairy roots
Source: BMC Plant Biol. 2014 Sep 16;14:245. doi: 10.1186/s12870-014-0245-z (PMC4172956; doi:10.1186/s12870-014-0245-z)

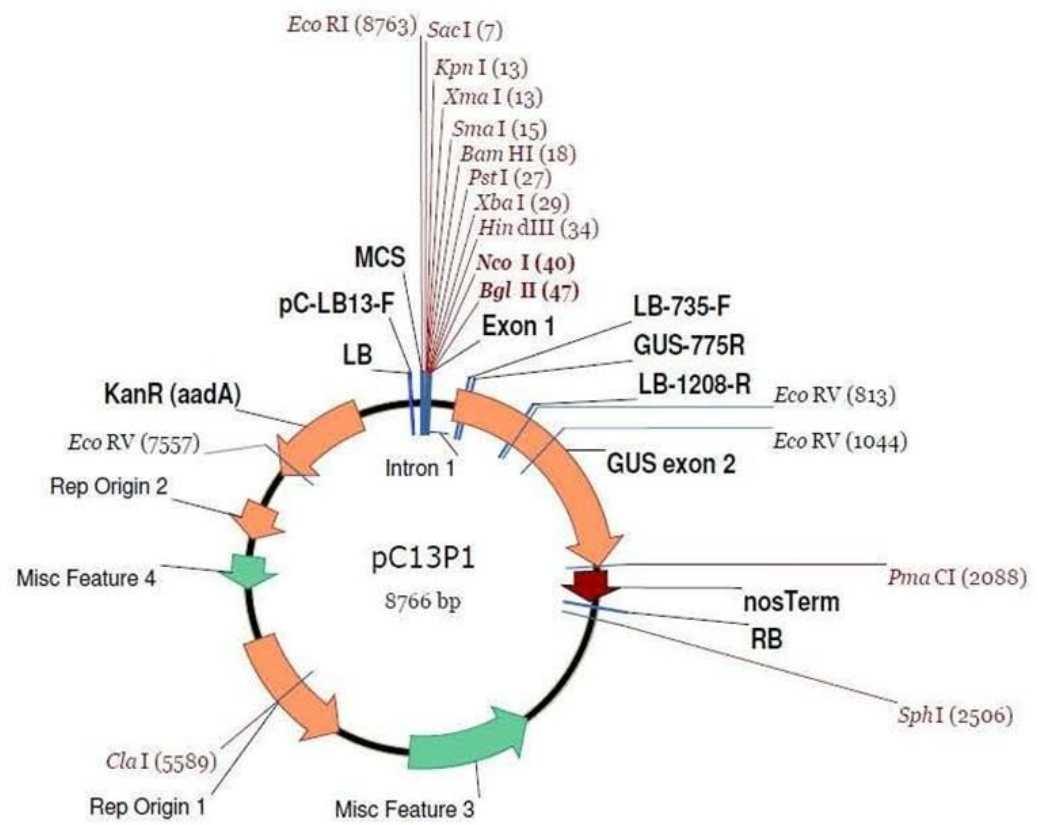

Supplement: Additional file 2: Figure S1. — The pC13P1 vector. [file 12870_2014_245_MOESM2_ESM.pdf]

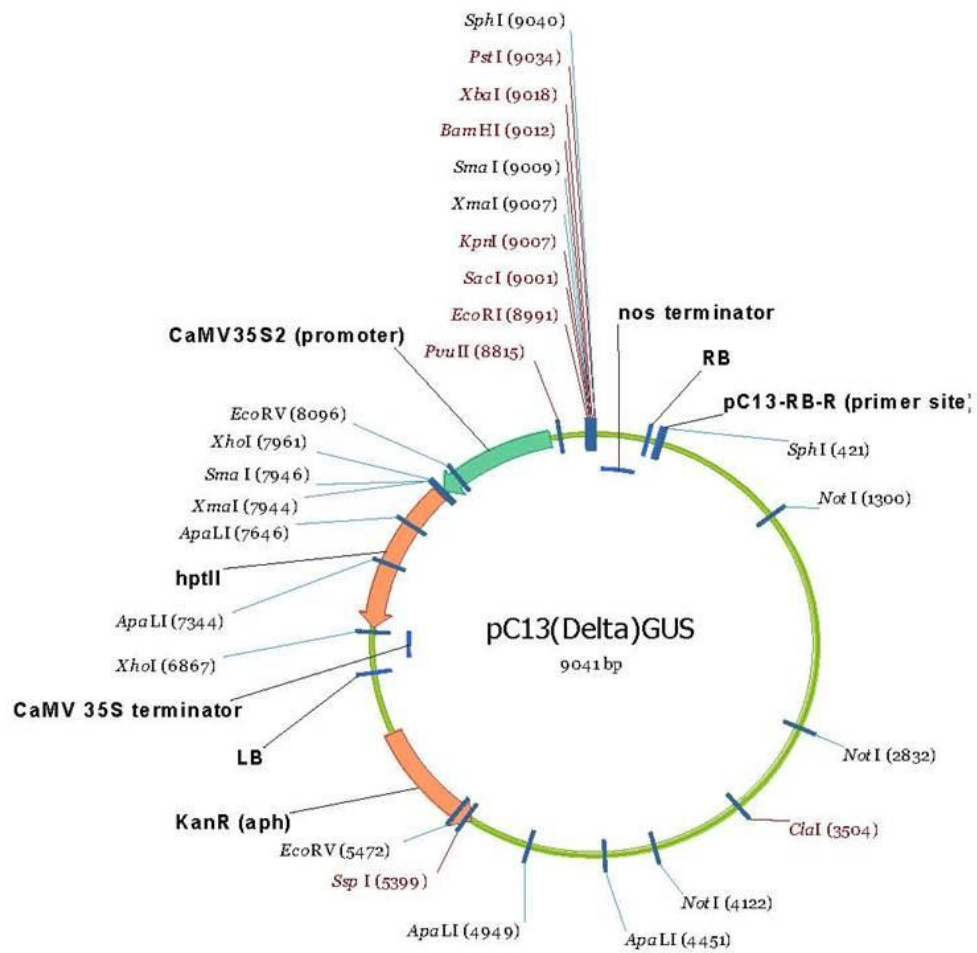

Supplement: Additional file 3: Figure S2. — The pC(Delt)GUS vector. [file 12870_2014_245_MOESM3_ESM.pdf]
